# Effects of lipids on the rate-limiting steps in the dark-to-light transition of Photosystem II core complex of *Thermostichus vulcanus*

**DOI:** 10.3389/fpls.2024.1381040

**Published:** 2024-03-21

**Authors:** Melinda Magyar, Parveen Akhtar, Gábor Sipka, Ildikó Domonkos, Wenhui Han, Xingyue Li, Guangye Han, Jian-Ren Shen, Petar H. Lambrev, Győző Garab

**Affiliations:** ^1^ Institute of Plant Biology, HUN-REN Biological Research Centre, Szeged, Hungary; ^2^ Photosynthesis Research Center, Key Laboratory of Photobiology, Institute of Botany, Chinese Academy of Sciences, Beijing, China; ^3^ Research Institute for Interdisciplinary Science, and Graduate School of Natural Science and Technology, Okayama University, Okayama, Japan; ^4^ Department of Physics, Faculty of Science, University of Ostrava, Ostrava, Czechia

**Keywords:** chlorophyll-a fluorescence, core complex of photosystem II, rate-limiting step, structural dynamics, thylakoid lipids, waiting time

## Abstract

In our earlier works, we have shown that the rate-limiting steps, associated with the dark-to-light transition of Photosystem II (PSII), reflecting the photochemical activity and structural dynamics of the reaction center complex, depend largely on the lipidic environment of the protein matrix. Using chlorophyll-*a* fluorescence transients (ChlF) elicited by single-turnover saturating flashes, it was shown that the half-waiting time (Δ*τ*_1/2_) between consecutive excitations, at which 50% of the fluorescence increment was reached, was considerably larger in isolated PSII complexes of *Thermostichus* (*T.*) *vulcanus* than in the native thylakoid membrane (TM). Further, it was shown that the addition of a TM lipid extract shortened Δ*τ*_1/2_ of isolated PSII, indicating that at least a fraction of the ‘missing’ lipid molecules, replaced by detergent molecules, caused the elongation of Δ*τ*_1/2_. Here, we performed systematic experiments to obtain information on the nature of TM lipids that are capable of decreasing Δ*τ*_1/2_. Our data show that while all lipid species shorten Δ*τ*_1/2_, the negatively charged lipid phosphatidylglycerol appears to be the most efficient species – suggesting its prominent role in determining the structural dynamics of PSII reaction center.

## 1 Introduction

Photosystem II (PSII) is a multi-subunit pigment-protein complex embedded in the thylakoid membranes (TMs) of plants, algae, and cyanobacteria. In nature, PSII core complexes (CCs) are present primarily in a dimeric form in the native TMs (Boekema et al., 1995; Kouril et al., 2012) but their monomeric forms can also be found (Takahashi et al., 2009; Watanabe et al., 2009). The main protein subunits of PSII CC are the reaction center (RC) proteins D1/D2, the inner antenna proteins CP43 and CP47, the α and β subunits of cytochrome *b_559_*, the oxygen-evolving complex (OEC), and a number of low molecular weight proteins (Umena et al., 2011). In addition, X-ray crystallography of PSII CC of *Thermostichus (T.) vulcanus* revealed approximately 20 lipids [6 monogalactosyldiacylglycerol (MGDG), 5 digalactosyldiacylglycerol (DGDG), 4 sulfoquinovosyldiacylglycerol (SQDG) and 5 phosphatidylglycerol (PG)] and at least 15 detergent molecules per monomer (Umena et al., 2011; Suga et al., 2015). The more recent cryo-EM structure of the same PSII CC solubilized with β-DDM – which possibly represents conditions closer to the physiological state – identified 18 of the lipids and 4 DDM molecules per monomer (Kato et al., 2021). The detergent molecules evidently replace different lipid molecules – and thus, might affect the photochemistry and structural dynamics of PSII.

PSII catalyzes the light-driven oxidation of water via capturing light energy by pigments situated in the antenna proteins, which is then transformed into electrochemical free energy within the RC complex (Cardona et al., 2012). When PSII is in dark-adapted open state (PSII_O_), upon illumination, an electron is transferred from the excited primary donor P_680_^*^ to the first electron acceptor pheophytin (Pheo), forming the P_680_^+^Pheo^−^ radical pair in several picoseconds [P_680_^*^ refers to a singlet excited state shared among several chlorins (Romero et al., 2017)]. Electron transfer steps then occur from Pheo^−^ to the first quinone electron acceptor, Q_A_, and from the tyrosine residue (Y_Z_) on the D1 protein to P_680_^+^. The final step, leading to the stabilization of the charge-separated state, is the oxidation of the Mn_4_CaO_5_ cluster. This state, with all Q_A_ reduced, is a closed state of PSII (PSII_C_), which is followed by somewhat slower, at physiological temperatures several hundred microseconds, electron-transfer steps between Q_A_ and the secondary quinone acceptor Q_B_. This step can be blocked by PSII inhibitor molecules, such as 3-(3′,4′ dichlorophenyl)-1,1′ dimethylurea (DCMU). In the presence of DCMU the lifetime of PSII_C_ becomes considerably longer, limited only by the charge recombination between Q_A_^−^ and S_2_^(+)^ of the OEC (Tyystjarvi and Vass, 2004).

In our earlier works, we recorded the *F*_v_ variable chlorophyll-*a* (Chl-*a*) fluorescence transients of PSII on DCMU-treated isolated plant TMs and PSII CC of *T. vulcanus* (*F*_v_ = *F*_m_ − *F*_o_, where *F*_m_ and *F*_o_ are the maximum and the minimum fluorescence levels, respectively; *F*_o_ is associated with PSII_O_). The technique of Chl-*a* fluorescence transient, also called ChlF, is one of the most commonly used tools to monitor the activity of oxygenic photosynthesis (Govindjee and Papageorgiou, 2004; Papageorgiou and Govindjee, 2011). We have shown, in accordance with Joliot and Joliot, 1979, that the fluorescence level of PSII_C_ after the first single turnover saturating flash (STSF) (with all Q_A_ reduced) display an *F*_1_ < *F*_m_ fluorescence yield, and that *F*_m_ can only be produced gradually by several (or at cryogenic temperatures by a large number of) STSFs; we have also discovered that the fluorescence increments from *F*_1_ to *F*_m_ require waiting times (Δ*τ*) between excitations, revealing rate limiting steps in the dark-to-light transition of PSII (Magyar et al., 2018). Further, we have shown that after the closure of PSII, by the first STSF, additional excitations produce only rapidly recombining P_680_^+^Pheo^–^ radical pairs (Sipka et al., 2019). The stepwise fluorescence rise of PSII, upon exposing PSII_C_ to a train of STSFs, has been shown to reflect the gradual formation of the light-adapted charge-separated state (PSII_L_), most probably driven by stationary and transient electric fields, generated by S_2_^(+)^Q_A_^−^ and P_680_^+^Pheo^–^, respectively, and dielectric relaxation processes (Sipka et al., 2021). Variable ChlF of closed PSII reaction centers, responsible for a substantial part of *F*_v_, has also been observed using long saturating light pulses and higher plant leaves (Laisk and Oja, 2020).

Our recent investigations revealed that the Δ*τ*_1/2_ half-waiting times, at which 50% of the *F*_k_-to-*F*_k+1_ (k = 1, 2, 3…) fluorescence increments are reached, depended strongly on factors affecting the rigidity of the samples. In particular, Δ*τ*_1/2_ was shown to sharply increase by lowering the temperature from 5°C to −80°C: from about 0.2 to 1 ms and from about 1 to 4 ms in DCMU-treated spinach TMs and PSII CC of *T. vulcanus*, respectively (Magyar et al., 2023). The difference between TMs and PSII CC was also observed in *T. vulcanus* cells compared to detergent-solubilized isolated PSII CC – pointing to the importance of the native RC environment in the TMs. Indeed, externally added TM lipids largely restored the short Δ*τ*_1/2_ observed in the cells, whereas non-TM lipids, which induced minor changes in the organization of PSII CC, exerted no effect on Δ*τ*_1/2_ (Magyar et al., 2022). These data strongly suggest the specific role of lipids in the structural dynamics of PSII.

Thylakoid membranes, on the one hand, serve as a matrix for photosynthetic complexes (Nelson and Ben-Shem, 2004). On the other hand, they play important role in the functioning and structure of membrane proteins through interactions between lipids and proteins embedded in the membrane (Mcintosh and Simon, 2006; Van Eerden et al., 2017). TMs contain two neutral lipids, MGDG and DGDG, accounting for ~40-50 and ~20-30 mol% of TM lipids and two negatively charged lipid species, SQDG (~10-30 mol%) and PG (5-15 mol%) (Murata et al., 1981; Dorne et al., 1990; Sakurai et al., 2006).

In a recent review of Yoshihara and Kobayashi (2022), the different lipid content of different PSII preparations from cyanobacteria, plants and algae were collected showing high diversity mostly in the amount of PG molecules. MGDG, the only non-bilayer lipid in the TM (Garab et al., 2022), has a special role, among others, in maintaining the activity of the embedded protein complexes (Jarvis et al., 2000; Jones, 2007; Kobayashi et al., 2013) and in dimerization of the monomeric PSII (Guskov et al., 2009; Kansy et al., 2014). Different treatments leading to the partial degradation of MGDG (Aronsson et al., 2008; Leng et al., 2008; Wu et al., 2013) showed only slight decrease in the oxygen-evolving activity of PSII, suggesting that the non-degraded fraction of MGDG is deeply buried in PSII and is responsible for retaining its structure and function (Yoshihara and Kobayashi, 2022). DGDG might be involved in the stabilization of the OEC and the assembly of extrinsic proteins (Sakurai et al., 2007). SQDG influences the electron transfer from OEC to Y_Z_ (Minoda et al., 2003), most probably due to its stabilizing effect on the binding of the manganese cluster and extrinsic proteins to PSII (Yoshihara and Kobayashi, 2022), and also impairs the Q_B_ exchange at the acceptor site (Nakajima et al., 2018). It was also shown in SQDG-deficient mutants in phosphate-starved conditions, where PG content is decreased, that SQDG acts as a substitute for anionic phospholipids (Güler et al., 1996; Yu et al., 2002). In the same mutants under phosphate-rich conditions, PG is shown to be able to replace SQDG and functions to support the PSII activity (Nakajima et al., 2018). PG was shown to play an important role in dimerization of PSII (Kruse et al., 2000; Sakurai et al., 2003) and the electron transfer at the Q_B_-binding site (Gombos et al., 2002; Sakurai et al., 2006; Leng et al., 2008).

The crystal structure of PSII CC of *T. vulcanus* (Umena et al., 2011; Suga et al., 2015) has revealed an asymmetric distribution of the lipid molecules – with MGDG (except one) and DGDG on the lumenal side of TM and with the headgroups of SQDG and PG facing the cytoplasmic side. Three lipids (one MGDG and two SQDGs) are situated at the monomer-monomer interface together with at least 6 detergent molecules, 13 lipids (3 MGDGs, 4 DGDGs, 1 SQDG, and 5 PGs) surround the D1/D2 heterodimer and 4 lipids (2 MGDGs, 1 DGDG, and 1 SQDG) are located in the periphery of PSII CC. Seven lipids (1 MGDG, 3 DGDGs, 1-1 SQDG and PG) are found between D1 and CP43 and 3 PGs are close to Q_A_ and one to Q_B_. It should be emphasized that the high flexibility and mobility of the lipid molecules makes their correct identification difficult in protein complexes, not to mention the perturbation caused by the detergents used for the sample preparation.

In this work, we investigated the role of different lipid molecules in determining the rate-limiting steps in the dark-to-light transition of DCMU-treated PSII CC of *T. vulcanus*. In particular, we determined Δ*τ*_1/2_ of ChlF in the absence and presence of different externally added TM lipid classes and their mixtures. Circular dichroism (CD) and 77 K fluorescence emission spectroscopy measurements were applied to test possible effects on the molecular organization of the complexes. We found that (i) while upon the addition of the externally added lipids, only minor differences were observed in the molecular architecture of PSII CC; (ii) the Δ*τ*_1/2_ values were about 45-50% shorter in the presence of the non-ionic lipids MGDG and DGDG; (iii) the negatively charged lipids, SQDG and PG, were more efficient, leading to ~55-60% shortening of Δ*τ*_1/2_, with PG being the most efficient lipid acting already at low concentrations. Our findings suggest that lipids near the RC chromophores act as mechanical transducers and play key roles in warranting the structural dynamics related to the dielectric relaxation processes associated with the PSII_C_-to-PSII_L_ transition.

## 2 Materials and methods

### 2.1 Source material

A thermophilic cyanobacterial strain, *T. vulcanus* was isolated from a hot spring in Yunomine, Japan (Koike and Inoue, 1983). Cells were grown photoautotrophically as batch culture in a BG11 medium (pH 7.0) at 50°C, and were continuously illuminated with a white fluorescent lamp at 50–100 μmol photons m^−2^ s^−1^ photon flux density (Shen et al., 2011). Cultures were aerated on a gyratory shaker operating at 100 rpm.

### 2.2 Sample preparation

PSII CCs of *T. vulcanus* were isolated as described earlier (Shen and Inoue, 1993; Shen and Kamiya, 2000; Shen et al., 2011; Kawakami and Shen, 2018). During the final steps of the purification, dimers and monomers were separated from crude PSII by an anion exchange column, with a column buffer (30 mM MES-NaOH (pH 6.0), 3 mM CaCl_2_, and 0.03% β-DDM) with a linear gradient of 170–300 mM NaCl. After collection of the monomer and dimer fractions PEG 1,450 was added at a final concentration of 13% to the samples which were then centrifuged to precipitate and concentrate them. Finally, they were suspended in a buffer containing 30 mM MES–NaOH (pH 6.0), 20 mM NaCl, and 3 mM CaCl_2_ and stored in liquid nitrogen or at -80°C until use. For spectroscopic measurements, isolated PSII CCs were diluted in a reaction buffer (5% glycerol, 20 mM MES (pH 6.0), 20 mM NaCl and 3 mM CaCl_2_).

For CD spectroscopy and fluorescence yield measurements PSII CCs were diluted to ~10 µg Chl mL^−1^ in the reaction buffer, and for fluorescence emission spectroscopy it was diluted to ~1 µg Chl mL^−1^. All experiments, except in section 3.1, were carried out on dimeric PSII CC. Stock solution of thylakoid lipids (MGDG, DGDG, SQDG and PG, Avanti Polar Lipids, Sigma) were prepared in methanol to a final concentration of 10 mg mL^−1^. These lipids, according to Sigma, contain predominantly unsaturated (18:3) fatty acids – as typical for higher plant TMs (Siegenthaler and Murata, 1998). The PSII CC suspension was mixed in the dark with lipids at different concentrations (with methanol not exceeding 0.8% final concentration) along with 40 µM DCMU, which was dissolved in dimethyl sulfoxide (with a final solvent concentrations of 1%).

### 2.3 Circular dichroism spectroscopy

CD spectra in the range of 350–750 nm were recorded at room temperature with a Jasco J-815 spectropolarimeter (Jasco, Japan). The measurements were performed in a semi-micro quartz cell of 1 cm optical path length with 2 nm spectral bandwidth.

### 2.4 Fluorescence yield measurements

Chl-*a* fluorescence yields were measured using a Multi-Color (MC) PAM (Walz, Effeltrich, Germany). Fluorescence increments were induced by STSFs (Xe flashes, Excelitas LS-1130-3 Flashpac with FX-1163 Flashtube with reflector, Wiesbaden, Germany) of 1.5-µs duration at half-peak intensity. The frequency of the low intensity and non-actinic modulated measuring light was 1 kHz. This restricted the time resolution of our ChlF transition measurements to several milliseconds. Earlier, using DCMU-treated PSII CC of *T. vulcanus* exposed to repetitive STSFs, we have identified the formation and rapid recombination of the P_680_^+^Pheo^-^ radical pair (Sipka et al., 2019). These recombination events evidently bring about, with a certain probability, the formation of triplet states, which under aerobic conditions relax in the time range of several microseconds (Lambrev et al., 2012; Laisk and Oja, 2020). These events do not destabilize the samples – as evidenced by double-STSF ChlF experiments presented in this paper, showing that doubling the flash intensity does not affect the fluorescence level at the millisecond time range [see also (Magyar et al., 2018; Sipka et al., 2021)].

The sample was placed on the sample holder of a thermoluminescence apparatus in order to control the temperature (Magyar et al., 2018). The timing of the flashes was controlled using a digital pulse generator (525 Six Channel Digital Pulse/Delay Generator, Berkeley Nucleonics, California, USA). The kinetic traces were recorded using MC-PAM’s own software.

The fluorescence transients were recorded after 10 min dark adaptation of the sample at room temperature, followed by an additional 5 min dark adaptation at 5°C, on which temperature all measurements were performed. At this temperature the recombination rate of the charge-separated state is very low, as reflected by thermoluminescence glow curves exhibiting the Q band above 20°C (Sipka et al., 2019); hence, allowing an easier determination of the magnitude of the Δ*τ*-dependent increments between the fluorescence levels elicited by the first and second STSFs (see the schematic representation of the measuring protocol in **Supplementary Animation S1**).

The *F*_1_-to-*F*_2_ Chl-*a* fluorescence increments were determined by measuring the fluorescence level after the second STSF, delivered by different waiting times (Δ*τ*) after the first flash, and were normalized to the *F*_m_ values, *i.e.*, the maximal fluorescence levels, which were determined using saturating multiple-turnover long laser flashes. The (*F*_2_-*F*_o_)/(*F*_m_-*F*_o_) values are plotted as a function of Δ*τ*. For simplicity, the first data points obtained after simultaneously firing the two STSFs are given at Δ*τ* = 10^-1^ µs. The Δ*τ*_1/2_ half-rise time values, *i.e.*, the Δ*τ* values at 50% of the maximum of the *F*_1_-to-*F*_2_ increments, were determined using a logistic fit function.

### 2.5 Steady-state fluorescence spectroscopy

Fluorescence emission spectra were measured at 77 K using an FP-8500 (Jasco, Japan) spectrofluorometer. Emission spectra in the range of 620–780 nm were recorded with excitation wavelength of 440 nm and excitation/emission bandwidth of 2.5 nm. The measurements were performed with 1 nm increments and 2 s integration time. Samples were cooled in a home-built accessory used with the FP-8500 spectrofluorometer. The spectra were corrected for the spectral sensitivity of the instrument using a calibrated light source (ESC-842, Jasco) as a reference.

### 2.6 Gel electrophoresis

The purity of PSII dimers and monomers was checked by blue native polyacrylamide gel electrophoresis on a 5–13% (w/v) gradient gel at 6°C according to (Schagger et al., 1994). The native gel was stained with Coomassie Blue G-250 to show all protein content of the samples. The amount of the samples loaded on each lane was 0.5 μg of Chl.

## 3 Results and Discussion

In order to elucidate which lipid(s) played a role in our earlier observed experiments affecting the Δ*τ*_1/2_ values (Magyar et al., 2022), we performed double-STSF induced Chl-*a* fluorescence measurements on PSII CC dimers mixed with various TM lipids in different Chl:lipid ratios. With these experiments we aimed to shed light on the nature of the ‘missing’ lipids (replaced by detergent) that might be responsible for the shorter half-waiting time in the native or TM-lipid reconstituted samples compared to the isolated PSII CC (cf. also Introduction).

### 3.1 Determination of Δ*τ*_1/2_ in monomers and dimers of PSII CC

**Figure 1:**
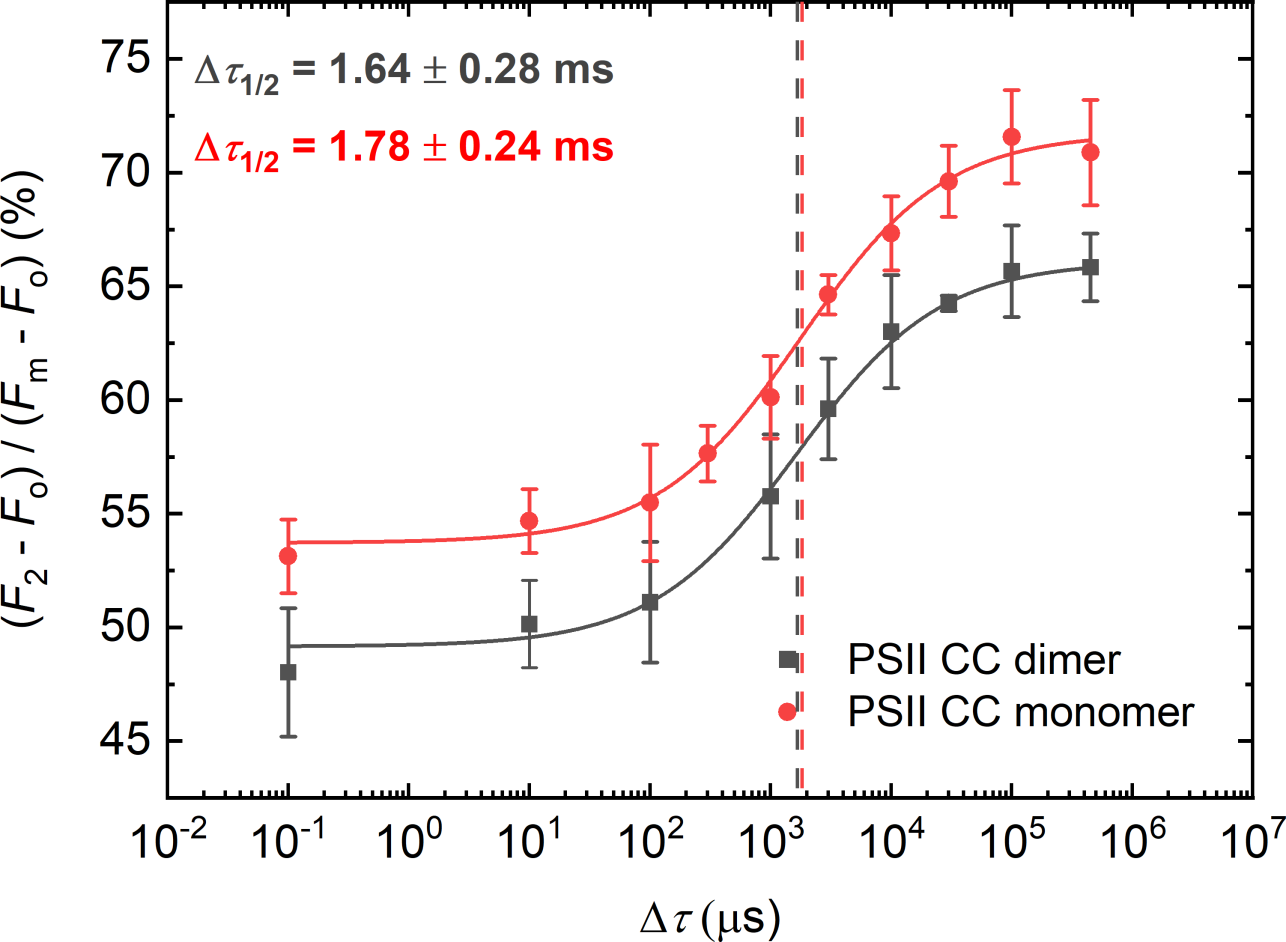
Dependence of the *F*_1_-to-*F*_2_ Chl-*a* fluorescence increments of DCMU-treated PSII CC of *T. vulcanus* dimer (black) and monomer (red) on the waiting time (Δ*τ*) between the first and the second STSF. Continuous lines represent logistic-function fits of the data points, mean values ± SD (*n* = 3–5). The vertical lines mark the Δ*τ*_1/2_ half-rise time values, *i.e.*, the Δ*τ* values at 50% of the maximum of the *F*_1_-to-*F*_2_ increments. For better visibility, the curve for the monomeric PSII CC was shifted upward by ~5% with respect to the starting points. Data points obtained with simultaneously fired STSFs are plotted at Δ*τ* = 10^-1^ µs.

As revealed by X-ray crystallography of PSII CC of *T. vulcanus*, detergent molecules tend to accumulate in domains interconnecting the two monomers in the dimer (5 detergent molecules per monomer) (Umena et al., 2011; Yu et al., 2021). Thus, we tested if isolated monomers exhibit larger Δ*τ*_1/2_ values than dimers – given their potentially greater exposure to detergents than in dimers. However, as shown in **Figure 1**, monomers and dimers display essentially the same half-waiting time values – rendering our hypothesis unlikely. These data are also consistent with our earlier finding that the stepwise Chl-*a* fluorescence rise, and the kinetics of the fast-fluorescence rise of monomeric and dimeric complexes are virtually indistinguishable (Sipka et al., 2021). Hence, the lipids ‘missing’ from the monomer-monomer interface are unlikely to be responsible for the increased Δ*τ*_1/2_ in PSII CC compared to the native systems (**Supplementary Material**, **Figure S1** confirms the oligomerization state of samples).

### 3.2 Effects of different lipid species on Δ*τ*_1/2_ of PSII CC

To test the effect of TM lipids separately on the rate-limiting step in the *F*_1_-to-*F*_2_ increment, we performed double-STSF induced fluorescence measurements on PSII CC of *T. vulcanus*, mixed with different TM lipids at different Chl:lipid ratios, and compared them with that of PSII CC dimers of the same batch. In a separate experiment, we checked that methanol – in which lipids were dissolved – exerted no effect at the given concentration (0.8% in the final volume) on Δ*τ*_1/2_. It displayed about the same half-waiting time, 1.5 ± 0.3 ms as the control, 1.6 ± 0.3 ms; methanol at 1.6% increased Δ*τ*_1/2_ to 3.51 ± 0.77 ms (**Supplementary Material**, **Figure S2**, **Supplementary Table S1**).

In earlier experiments (Magyar et al., 2022), PSII CCs were reconstituted into liposomes composed of TM lipids in proportions as in plants: 45% (w/v) MGDG, 30% DGDG, 15% PG, and 10% SQDG; at a final Chl:lipid ratio of 1:8. Here, we performed experiments to test the effects of individual lipid head group species on Δ*τ*_1/2_, using the same batch of PSII CC dimers. In this batch, the minimum Δ*τ*_1/2_ value of PSII CC embedded in liposomes was 0.5 ± 0.1 ms (**Supplementary Material**, **Table S1**); this value is somewhat larger than in the native TMs and in the batch of PSII CC used earlier (Magyar et al., 2022). This suggests batch-to-batch variation in the lipid content of the isolated PSII CCs and/or in their capability of replenishing the missing lipid molecules.

**Figure 2:**
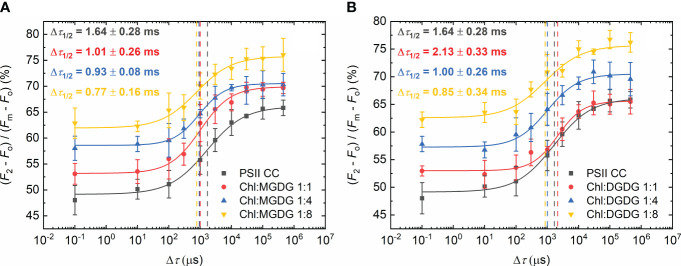
Dependence of the *F*_1_-to-*F*_2_ Chl-*a* fluorescence increments of DCMU-treated PSII CC of *T. vulcanus* on the waiting time (Δ*τ*) between the first and the second STSF in the absence (PSII CC) and presence of externally added MGDG **(A)** and DGDG **(B)** at Chl:lipid ratios as indicated. Continuous lines represent logistic-function fits of the data points, which represent mean values ± SD (*n* = 3–5). Dotted vertical lines mark the Δ*τ*_1/2_ half-rise time values. For better visibility, each curve was shifted upward by ~5% with respect to the previous one.

In the following series of experiments, we added different isolated lipids and used different Chl:lipid ratios to test their effect on Δ*τ*_1/2_. The two non-ionic lipids, MGDG and DGDG exerted similar effects on the half-waiting time, which displayed slightly decreasing patterns towards higher lipid concentrations (**Figure 2**). Upon gradually increasing the concentration of the externally added MGDG, from 1:0 to 1:8 Chl:lipid ratio, Δ*τ*_1/2_ of 1.6 ± 0.3 ms in the control gradually decreased to 0.8 ± 0.2ms (**Figure 2A**), indicating that more MGDG might be needed to occupy places in PSII CC responsible for the decrease of Δ*τ*_1/2_ in TMs. DGDG showed almost identical parameters with the exception of the measurement with the Chl:lipid ratio of 1:1, where Δ*τ*_1/2_ increased to 2.1 ± 0.3 ms (**Figure 2B**); these data suggest that DGDG has low ability to replace the detergent molecules around the site regulating the rate-limiting steps of PSII. Nonetheless, when applied at higher concentrations, it was capable of decreasing Δ*τ*_1/2_. It is to be noted that the slope (*P*) of the fitted curves became slightly steeper after the addition of these lipids (**Supplementary Table S1**), a phenomenon which requires further investigation.

**Figure 3:**
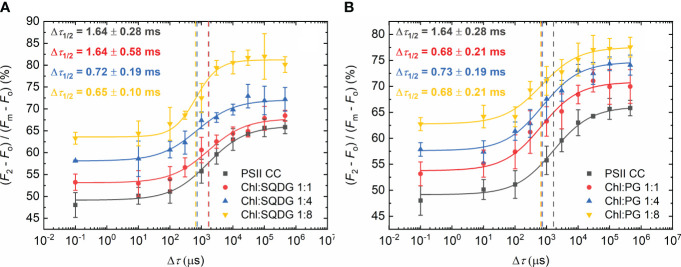
Dependence of the *F*_1_-to-*F*_2_ Chl-*a* fluorescence increments of DCMU-treated PSII CC of *T. vulcanus* on the waiting time (Δ*τ*) between the first and the second STSF in the absence (PSII CC) and presence of externally added SQDG **(A)** and PG **(B)** at Chl:lipid ratios as indicated. Continuous lines represent logistic-function fits of the data points, which represent mean values ± SD (*n* = 3–5). Dotted vertical lines mark the Δ*τ*_1/2_ half-rise time values. For better visibility, each curve was shifted upward by ~5% with respect to the previous one.

When the negatively charged lipids SQDG and PG were added, a larger decrease of the half-waiting times was observed (**Figure 3**). With SQDG, this occurred only after applying 1:4 Chl:lipid ratio, reaching a value of 0.7 ± 0.2 ms (**Figure 3A**). In contrast, PG shortened Δ*τ*_1/2_ immediately after the addition of only one PG molecule per Chl, reaching about the same Δ*τ*_1/2_ value of 0.7 ± 0.2 ms, and no further decrease was attained by additional PG molecules (**Figure 3B**). It is also to be noted that in this case, the *P* values remained almost the same as in PSII CC (**Supplementary Table S1**).

### 3.3 Effects of different lipid mixtures on Δ*τ*_1/2_ of PSII CC

**Figure 4:**
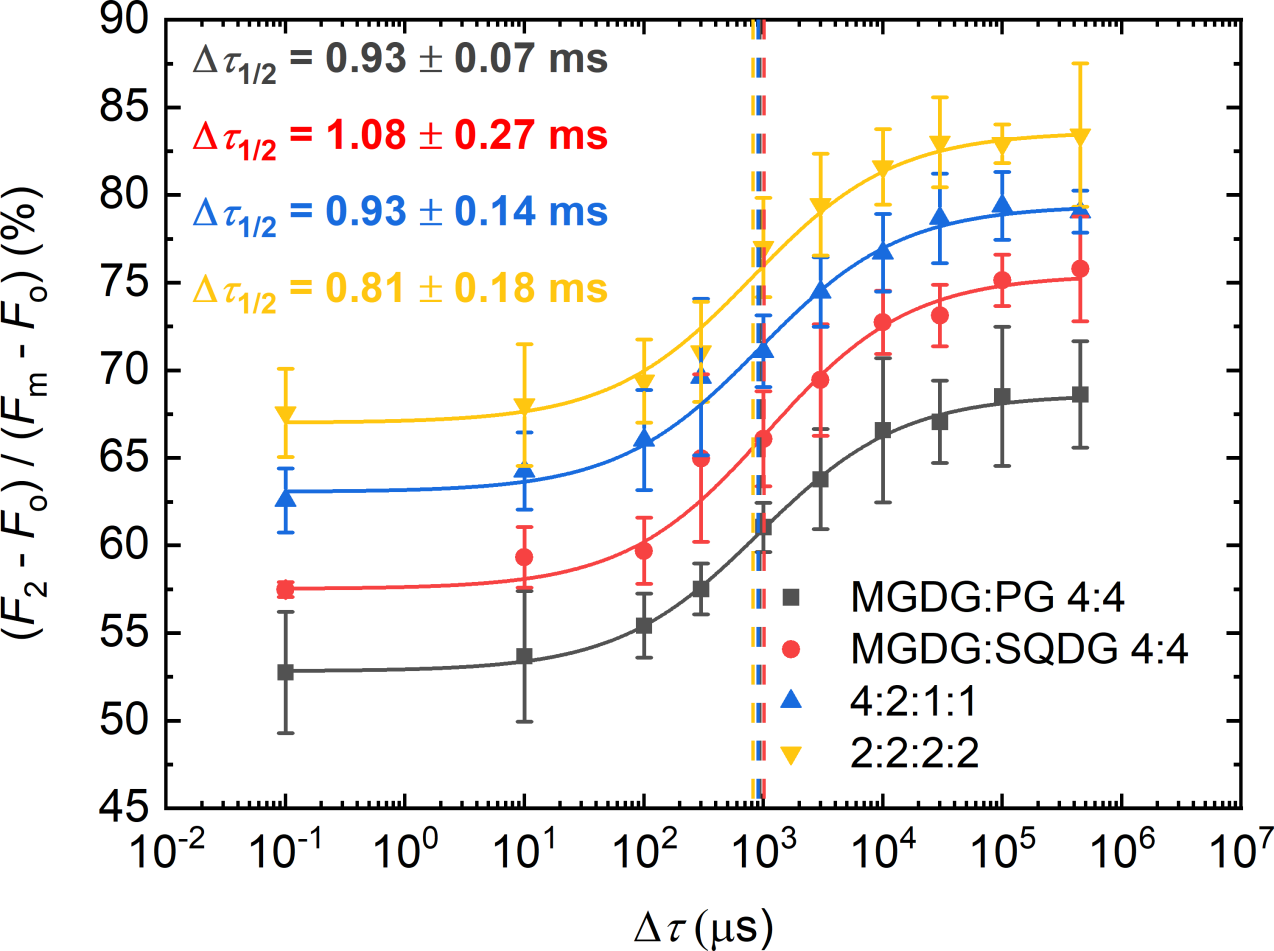
Dependence of the *F*_1_-to-*F*_2_ Chl-*a* fluorescence increments of DCMU-treated PSII CC of *T. vulcanus* on the waiting time (Δ*τ*) between the first and the second STSF in the presence of different concentrations of externally added lipid mixtures of two or four different lipids, MGDG : PG and MGDG : SQDG or MGDG : DGDG : SQDG : PG, respectively, in proportions as indicated, at Chl:lipid ratio of 1:8. Continuous lines represent logistic-function fits of the data points, mean values ± SD (*n* = 3–5). The vertical lines mark the Δ*τ*_1/2_ half-rise time values. For better visibility each curve was shifted upward by ~5% with respect to the previous curve.

In these experiments, TM lipid molecules were added to PSII CC in different mixtures and proportions, keeping the same Chl:lipid ratio of 1:8 (**Figure 4**). It was interesting to observe that PG and SQDG when combined with MGDG were less efficient than when they were applied alone (displaying Δ*τ*_1/2_ values of 0.93 ± 0.07 ms and 1.08 ± 0.27 ms *vs.* about 0.7 ms). It seems that in each case, the most abundant TM lipid species, MGDG, hindered rather than facilitated the PG-induced shortening of Δ*τ*_1/2_ – most probably because of competing with PG and/or SQDG to replace the detergent and occupy the space of the missing lipids. The same appeared to be true for DGDG. With lipid mixtures of MGDG : DGDG : SQDG : PG of 4:2:1:1, which is close to the lipid-species ratio of plant TMs, Δ*τ*_1/2_ remained about the same as in the case of MGDG : PG. Further decrease was achieved when all lipids were present in equal concentrations (MGDG : DGDG : SQDG : PG of 2:2:2:2). In this case, the half-waiting time value (0.8 ± 0.1 ms) assumed almost the same value as with PG alone.

The observation that PG seemed to be more effective than SQDG, suggests that PG is the most important ‘missing’ lipid from isolated PSII CC. There are indications in the literature that lipids can exchange each other (Güler et al., 1996; Yu et al., 2002; Kansy et al., 2014), thus, as both of these lipids are negatively charged, it is highly likely that in the absence of additional PG, SQDG might occupy its place. It must also be taken into account that non-ionic lipid molecules might have been replaced by anionic ones or *vice versa*; however, because of the different head groups of the lipids, this scenario is highly unlikely, unless these lipids are bound to PSII through hydrophobic interactions and not by ligation. It can be envisioned that excess amounts of the negatively charged lipids SQDG and PG replace neutral lipids at the binding sites both in the core-antenna complexes CP43 and CP47 and the D1/D2 heterodimer – which thus may influence the configuration of the local electric fields. Kansy et al. (2014) suggested similar changes owing to the negatively charged lipids with the possible explanation of the dissociation of CP43.

### 3.4 Spectral changes induced by the addition of lipids

**Figure 5:**
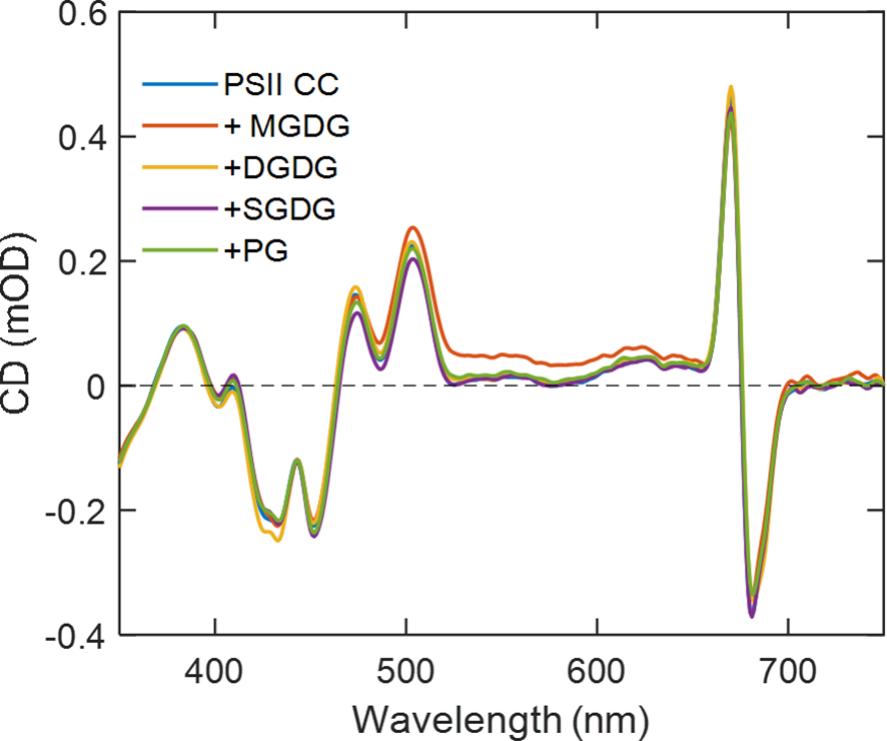
Room-temperature CD spectra of *T. vulcanus* PSII CCs in the absence and presence of different TM lipids added externally at Chl:lipid ratio of 1:8. The spectra are normalized to unity absorbance of each sample at 675 nm.

CD spectra of PSII in the visible wavelength region specifically probe excitonic couplings between pigments in the CC. No specific changes in the CD spectra of the PSII CC were observed upon the addition of either of the four thylakoid lipids, except for minor deviations that could be attributed to baseline and differential light scattering effects due to the sample turbidity in the presence of lipids (**Figure 5**). These data are in good agreement with the absence of detectable changes in the CD spectra of PSII CC in solution and in reconstituted lipid membranes (Magyar et al., 2022).

**Figure 6:**
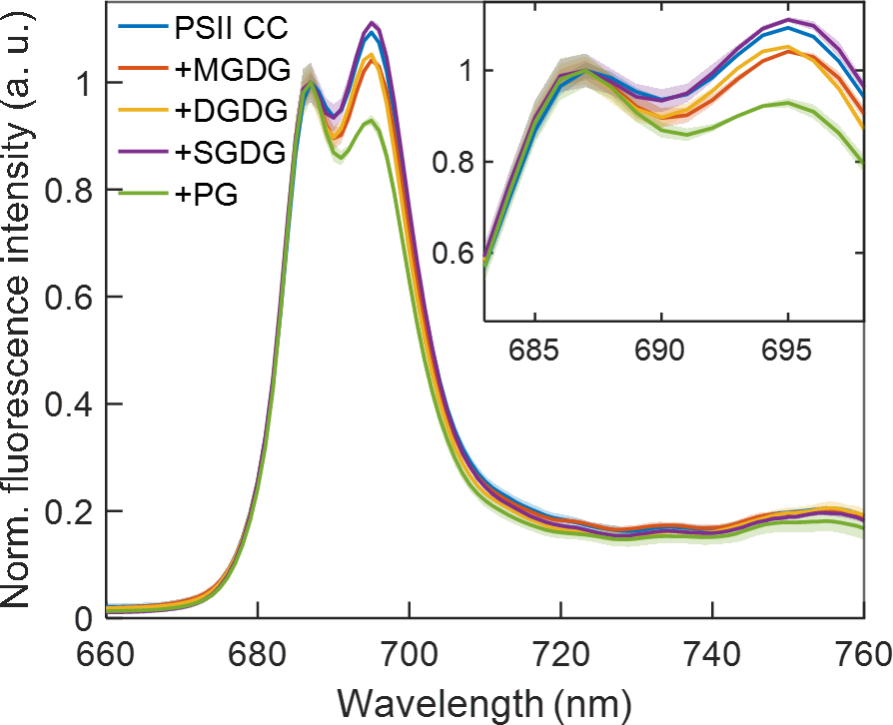
77 K fluorescence emission spectra of *T. vulcanus* PSII CC in the absence and presence of different TM lipids added externally at Chl:lipid ratio of 1:8 and excited at 440 nm. The spectra are normalized at 687 nm and are corrected for the detector spectral sensitivity. The spectra are average of six independent measurements, and the shaded area represents the standard deviation.

Previously we have shown that reconstitution of PSII CC in liposomes prepared with a thylakoid lipid mixture brought about significant changes in the fluorescence emission spectra recorded at 77 K (Magyar et al., 2022). Particularly, the emission peak at 687 nm gained intensity relative to the 694 nm peak upon insertion of PSII in the lipid membranes. These bands originating from CP43 and CP47, respectively are denoted F685 and F695 (Andrizhiyevskaya et al., 2005). We observed the same qualitative change upon adding different thylakoid lipids to PSII CC (**Figure 6**), which might originate from replacement of some of the detergent molecules in the periphery of CC (Umena et al., 2011; Suga et al., 2015). It is interesting to note that the changes were markedly different depending on the class of lipids used. SQDG exerted no significant effect on the fluorescence spectrum, whereas MGDG and DGDG decreased the relative intensity of the 694 nm peak. The negatively charged phospholipid PG had the strongest effect suppressing the emission at 694 nm relative to the short-wavelength band. Qualitatively, the spectral changes induced by the addition of lipids, especially PG, could be related to the difference in the fluorescence emission spectra of monomeric PSII CC compared to dimeric CC (**Supplementary Figure S3**). The similarity might suggest that the lipids destabilize the PSII dimers. The changes in both cases are consistent with the hypothesis that structural destabilization of the CC exposing the red-shifted Chls in CP47 to a more polar environment alters their excited-state energy distribution, in effect decreasing the fraction of CC complexes possessing F695 states.

### 3.5 The possible sites of ‘missing’ lipids participating in governing the rate-limiting steps

In our earlier study we showed that externally added lipids accelerated the rate of the PSII_C_-to-PSII_L_ transition (Magyar et al., 2022), and they were proposed to reoccupy the sites taken by detergent molecules.

**Figure 7:**
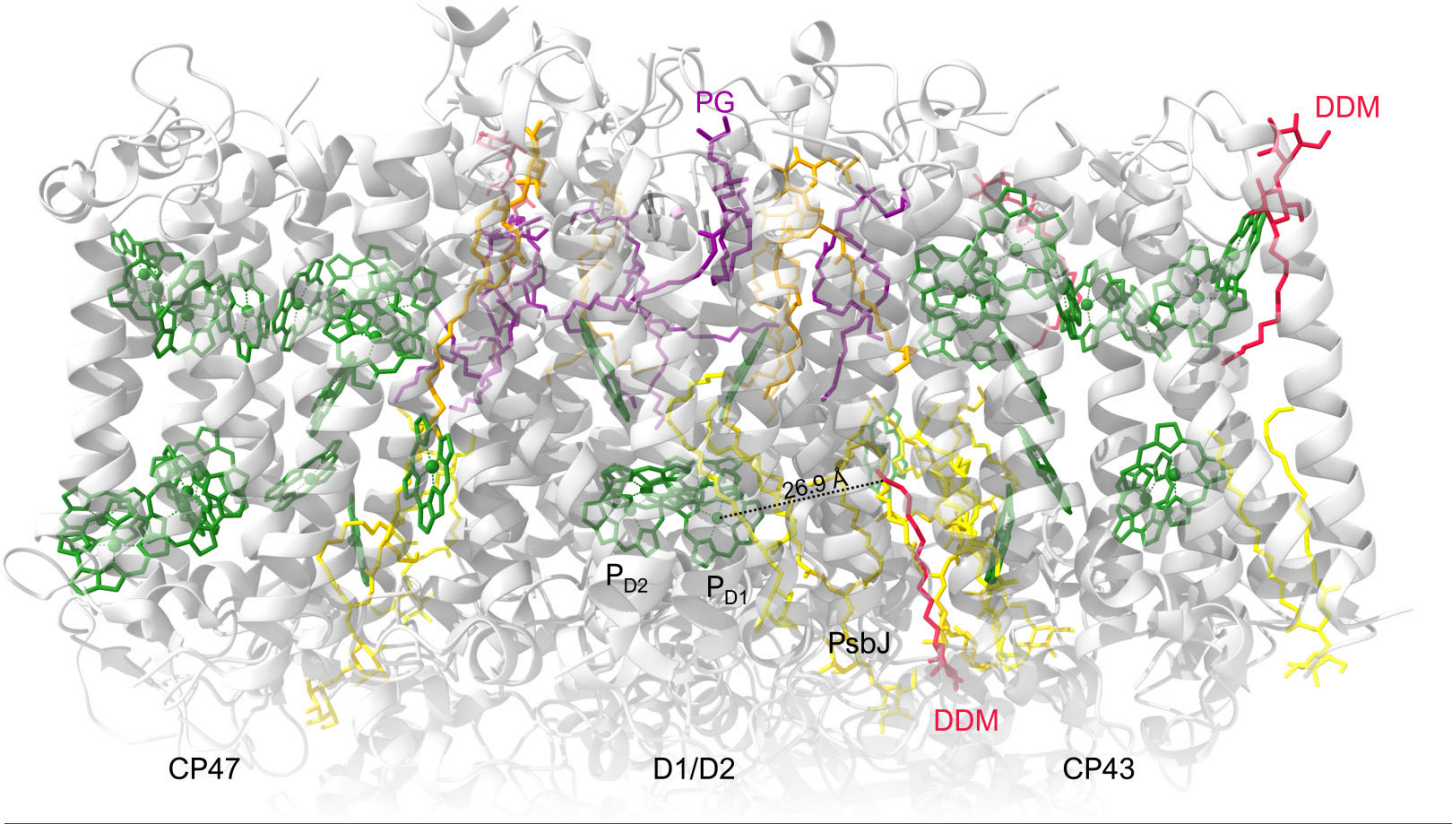
Overall cryo-EM structure of cofactors, detergents and lipids in PSII monomer from *T. vulcanus* (PDB: 7D1U). View of the PSII monomer perpendicular to the membrane normal. Chls of the RC and the core-antenna complexes (CP43 and CP47) are displayed in green. Color codes for lipids: MGDG, yellow; DGDG, gold; SQDG, orange; PG, dark magenta. Detergents are dark red.

By using the cryo-EM structure of PSII CC in β-DDM obtained by Kato et al. (2021), we can try to identify possible lipid-binding sites occupied by detergent molecules that may be influencing the PSII_C_-to-PSII_L_ transition dynamics (**Figure 7**). As pointed out above (Section 3.1), the ‘missing’ lipids, replaced by detergents, in the monomer-monomer interface are highly unlikely to affect the rate-limiting steps in the PSII_C_-to-PSII_L_ transition, as characterized by Δ*τ*_1/2_. Also, as proposed in Section 3.4, detergent molecules at the periphery of the CC, in the vicinity of CP43 and CP47 might contribute to the observed lipid addition-dependent changes in the 77 K emission spectra; however, these effects were not correlated with the lipid-induced shortening of Δ*τ*_1/2_.

A possible explanation of our observations is that reoccupying possible sites by lipids facilitates the light-induced reorganizations and shorten Δ*τ*_1/2_. Based on the scheme explaining the de-excitation pathways in PSII CC (Shibata et al., 2013) and the proposed mechanism involving the effects of prominently strong stationary and transient electric fields around the RC complex (Sipka et al., 2021), it is assumed that the site of the ‘missing’ and replenishable lipids are to be found near the cofactors of the RCs. For example, there is a DDM molecule interacting at about 27 Å distance from the primary donors that could be a possible candidate (**Figure 7**). It is in close contact with native lipids filling a gap between the D1 protein and the peripheral low-molecular-mass (LMM) subunits, in close contact with PsbJ. If this binding site influences the structural reorganizations of PSII, it will raise the question of the role of the LMM subunits, particularly PsbJ, in the light adaptation process. However, it cannot be ruled out that other sites not identified in the structure are responsible for the lipid/detergent exchange effects on the light-induced reorganizations.

According to an alternative, non-conflicting explanation, the externally added lipids surrounding PSII CC can prevent or reduce the probability of dissociation of structural lipids in the vicinity of the RC. It is very likely that association and dissociation of lipids in the protein complexes depend on the lipidic environment of PSII CC. In the absence of externally added lipids, some of the lipid molecules of PSII CC may gradually dissociate from the protein complex and might be released into the detergent solution. Such a mechanism might affect the binding of the two PG molecules LHG410 and LHG627 which have their fatty acyl chains located close to the primary donor Chl molecules, at a distance of 12 and 16 Å, respectively (Kato et al., 2021). Restoring the molecular environment around the primary donor can thus also be responsible for the observed exogenous-lipid dependent decrease of Δ*τ*_1/2_.

In general, lipids, because of their high structural flexibility, may act as mechanical transducer molecules facilitating reorganizations in the matrix of RC. The fact that the negatively charged lipid, PG, appears to be the most efficient in accelerating Δ*τ*_1/2_ might indicate that electrostatic interactions play important roles in the assembly and structural dynamics of the RC complex of PSII.

## 4 Conclusions

The aim of this work was to gain additional information on the roles of different TM lipids in the structural dynamics of PSII CC of *T. vulcanus*, as reflected by the Δ*τ*_1/2_ half-waiting times in the STSF-induced Chl-*a* fluorescence transients. Data obtained here – showing that each externally added TM lipid and their mixtures shorten Δ*τ*_1/2_ – are in good agreement with our earlier findings, which revealed that the half-waiting times were shorter in *T. vulcanus* cells and liposome-embedded PSII CCs than in isolated PSII CC (Magyar et al., 2022).

The shortening of Δ*τ*_1/2_ upon the addition of all TM lipid molecules show that replenishing the ‘missing’ lipids facilitates the PSII_C_-to-PSII_L_ transition – indicating that incorporation of lipids in PSII CC, most probably leads to replacing some of the detergent-occupied sites by lipid molecules. ‘Missing’ lipids, replaced by detergents, are found at the interface between the two monomers and in the core-antenna complexes (5 and 8 detergent molecules per monomer); the remaining molecules are located near the cofactors of the RC complex. As an alternative explanation, externally added lipids can be proposed to affect the association and dissociation of two PG molecules in the close vicinity of the Chl molecules in the RC (Suga et al., 2015; Kato et al., 2021; Yu et al., 2021). In this context it is also worth noting that in *T. vulcanus* the fatty acid composition of the different lipids is quite similar to each other, in PSII CC dominated by 16:0 (>50%) and 18:1 (15-27%) (Sakurai et al., 2006). In contrast, the fatty acids of the externally added plant TMs are highly unsaturated – the influence of which on the restoration of Δ*τ*_1/2_ remains to be clarified.

Spectroscopic data and analyses of the specificity of effects of different lipid species as well as considerations of the structure of PSII CC of *T. vulcanus* strongly suggest that lipid-replacements of detergents in the monomer-monomer interface of the dimer and/or in the core antenna complexes affect the excitation distribution at 77 K but are unlikely to shorten the Δ*τ*_1/2_ values. Decreasing the half-waiting time is most likely caused by reoccupation and/or closer association of the lipid binding sites near the RC complex, which thus partially restores the structural dynamics of PSII RCs. It appears that each lipid is capable, albeit with different affinities, to decrease the rate limiting steps in the PSII_C_-to-PSII_L_ transition – suggesting their role as mechanical transducers sensitive to the generation of the intense transient electric field due to the P_680_^+^Pheo^−^ radical pair and/or its rapid recombination generating fast local heat jumps [cf. Sipka et al. (2021)]. Further experiments, on reconstituted systems and/or on different wild-type or mutant PSII CCs are required to better understand the role of lipids in the structural dynamics of PSII RC.

## Data availability statement

The original contributions presented in the study are included in the article/**Supplementary material**. Further inquiries can be directed to the corresponding author.

## Author contributions

MM: Conceptualization, Writing – original draft, Data curation, Formal analysis, Investigation, Visualization, Writing - review & editing. PA: Writing – original draft, Data curation, Formal analysis, Funding acquisition, Investigation, Visualization. GS: Writing - review & editing, Methodology, Visualization. ID: Writing – review & editing, Investigation, Visualization. WH: Writing – review & editing, Resources. XL: Writing – review & editing, Resources. GH: Writing – review & editing, Resources. JS: Writing – review & editing, Funding acquisition, Resources, Supervision. PL: Conceptualization, Writing – review & editing, Formal analysis, Funding acquisition, Software, Supervision, Visualization. GG: Conceptualization, Writing – original draft, Funding acquisition, Methodology, Supervision, Writing – review & editing.
