# Supplementary material for: Effects of lipids on the rate-limiting steps in the dark-to-light transition of Photosystem II core complex of Thermostichus vulcanus
Source: Front Plant Sci. 2024 Mar 21;15:1381040. doi: 10.3389/fpls.2024.1381040 (PMC10991767; doi:10.3389/fpls.2024.1381040)
Supplement: Supplementary file 1 [file DataSheet_1.docx]

Supplementary Material


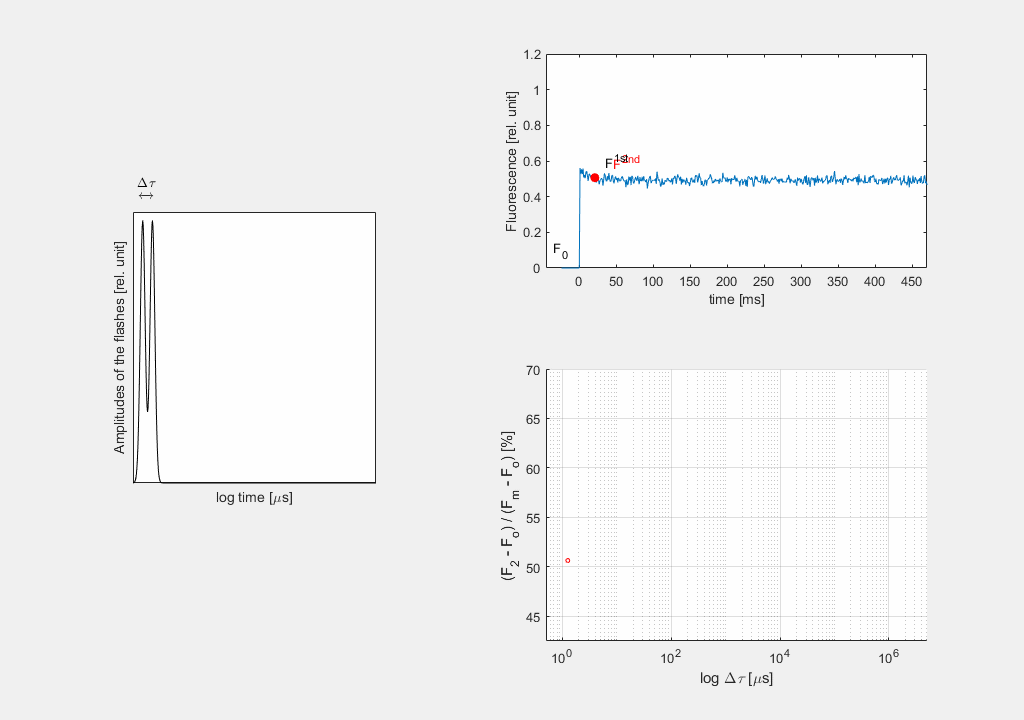


**Supplementary Animation S1:** Schematic representation of the double-STSF induced ChlF transient measurements. Two STSFs are fired with variable, discrete Δ*τ* time intervals between the first and the second flash (lhs panel). In each experiment, the *F*_2_ fluorescence level is determined after the second STSF (*F*^2nd^) and plotted as a function of Δ*τ* (rhs panels). The experimentally determined fluorescence levels are normalized to *F*_o_ and *F*_m_ ; in each experiment, *F*_m_ is determined by using an additional train of STSFs and/or an MTSF (not shown). The continuous curve in the lower rhs panel shows a typical logistic function fit obtained for DCMU-treated PSII CC measured at 5 °C (as in Fig. 1, logarithmic scale); the scatter of points in the lower panel (belonging to the fitted curve, they do not indicate data points), and noise in the upper panel are generated by software. The upper panel also emulates that the *F*_1_ level, generated solely by the first STSF (*F*^1st^), is not resolved with Δ*τ* ≤2 ms because of the selected time resolution of the detector unit; note the linear time-scale in this panel.


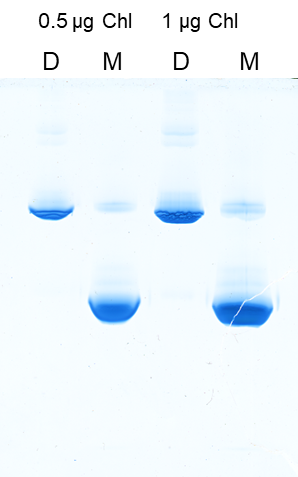


**Supplementary Figure S1.** BN-PAGE profiles of isolated PSII complexes from *T. vulcanus*. 0.5 µg chlorophyll was loaded for each sample on a linear 5-13% acrylamide gradient gel. The native gel was stained with Coomassie Blue G-250. D and M, respectively, samples of dimeric and monomeric complexes of PSII.

**Supplementary Figure S2.** Dependence of the *F*_1_-to-*F*_2_ Chl-*a* fluorescence increments of DCMU-treated PSII CC of *T. vulcanus* on the waiting time (Δ*τ*) between the first and the second STSF in the absence (PSII CC) and presence of externally added methanol at concentrations as indicated. Continuous lines represent logistic-function fits of the data points, which represent mean values ± SD (*n* = 3–5). Dotted vertical lines mark the Δ*τ*_1/2_ half-rise time values. For better visibility, each curve was shifted upward by ~5% with respect to the previous one.

**Supplementary Figure S3.** 77 K fluorescence emission spectra of *T. vulcanus* PSII dimers and monomers recorded with excitation wavelength of 440 nm. The spectra are normalized to maximum fluorescence intensities and are corrected for the spectral sensitivity of the system.

**Supplementary Table S1.** Double-STSF induced Δ*τ*_1/2_ half-rise times of DCMU-treated PSII CC of *T. vulcanus* mixed with different lipids in different Chl:lipid ratios and embedded in plant TM liposome; the values were obtained from experiments displayed in Figures 1-4. Measurements at 5 °C were performed on the same PSII CC batch of dimers; for comparison, we also show data obtained on monomers. Logistic fit *P* indicates the slope of the rise curve calculated by logistic-function fit of the data points, mean values ± SD (*n* = 3–5). Numbers marked with * are obtained from a global fit with shared *P* = 0.73; where the global fit was not satisfactory, we allowed free run of the fit. The *F*_v_/*F*_m_ parameter values are also shown.

|  | **Δ*τ*_1/2_ (ms)** | **Logistic fit *P*** | ***F*_v_/*F*_m_** |
| --- | --- | --- | --- |
| PSII CC monomer | 1.78 ± 0.24 | 0.73* | 0.75 ± 0.01 |
| PSII CC dimer | 1.87 ± 0.57  1.64 ± 0.28 | 0.73*  0.73* | 0.76 ± 0.02  0.77 ± 0.01 |
| 1.6% methanol  0.8% methanol | 3.51 ± 0.77  1.53 ± 0.31 | 0.73*  0.73* | 0.78 ± 0.01  0.76 ± 0.01 |
| Chl:MGDG = 1:1  1:4  1:8 | 1.01 ± 0.26  0.93 ± 0.08  0.77 ± 0.16 | 0.89  1.04  0.77 | 0.67 ± 0.02  0.61 ± 0.01  0.53 ± 0.01 |
| Chl:DGDG = 1:1  1:4  1:8 | 2.13 ± 0.33  1.00 ± 0.26  0.85 ± 0.34 | 1.07  0.88  0.73* | 0.70 ± 0.01  0.69 ± 0.01  0.69 ± 0.01 |
| Chl:SQDG = 1:1  1:4  1:8 | 1.64 ± 0.58  0.72 ± 0.19  0.65 ± 0.11 | 0.73*  0.73*  1.2 | 0.74 ± 0.02  0.63 ± 0.02  0.47 ± 0.03 |
| Chl:PG = 1:1  1:4  1:8 | 0.68 ± 0.21  0.73 ± 0.19  0.68 ± 0.21 | 0.73*  0.73*  0.73* | 0.71 ± 0.03  0.69 ± 0.04  0.65 ± 0.02 |
| Chl:MGDG:PG = 1:4:4 | 0.93 ± 0.07 | 0.73* | 0.68 ± 0.01 |
| Chl:MGDG:SQDG = 1:4:4 | 1.08 ± 0.27 | 0.73* | 0.73 ± 0.01 |
| Chl:MGDG:DGDG:SQDG:PG = 1:4:2:1:1 | 0.93 ± 0.14 | 0.73* | 0.73 ± 0.01 |
| Chl:MGDG:DGDG:SQDG:PG = 1:2:2:2:2 | 0.81 ± 0.10 | 1.01 | 0.71 ± 0.01 |
| Plant TM liposome | 0.49 ± 0.10 | 1.35 | 0.51 ± 0.03 |
